# Tyrosine Sulfation of Native Mouse Psgl-1 Is Required for Optimal Leukocyte Rolling on P-Selectin In Vivo

**DOI:** 10.1371/journal.pone.0020406

**Published:** 2011-05-25

**Authors:** Andrew D. Westmuckett, Kelly M. Thacker, Kevin L. Moore

**Affiliations:** 1 Cardiovascular Biology Research Program, Oklahoma Medical Research Foundation, University of Oklahoma Health Sciences Center, Oklahoma City, Oklahoma, United States of America; 2 Department of Cell Biology, University of Oklahoma Health Sciences Center, Oklahoma City, Oklahoma, United States of America; 3 Department of Medicine, University of Oklahoma Health Sciences Center, Oklahoma City, Oklahoma, United States of America; 4 Oklahoma Center of Medical Glycobiology, University of Oklahoma Health Sciences Center, Oklahoma City, Oklahoma, United States of America; Heart Center Munich, Germany

## Abstract

**Background:**

We recently demonstrated that tyrosine sulfation is an important contributor to monocyte recruitment and retention in a mouse model of atherosclerosis. P-selectin glycoprotein ligand-1 (Psgl-1) is tyrosine-sulfated in mouse monocyte/macrophages and its interaction with P-selectin is important in monocyte recruitment in atherosclerosis. However, whether tyrosine sulfation is required for the P-selectin binding function of mouse Psgl-1 is unknown. Here we test the function of native Psgl-1 expressed in leukocytes lacking endogenous tyrosylprotein sulfotransferase (TPST) activity.

**Methodology/Principal Findings:**

Psgl-1 function was assessed by examining P-selectin dependent leukocyte rolling in post-capillary venules of C57BL6 mice transplanted with hematopoietic progenitors from wild type (WT→B6) or *Tpst1;Tpst2* double knockout mice (*Tpst* DKO→B6) which lack TPST activity. We observed that rolling flux fractions were lower and leukocyte rolling velocities were higher in *Tpst* DKO→B6 venules compared to WT→B6 venules. Similar results were observed on immobilized P-selectin in vitro. Finally, *Tpst* DKO leukocytes bound less P-selectin than wild type leukocytes despite equivalent surface expression of Psgl-1.

**Conclusions/Significance:**

These findings provide direct and convincing evidence that tyrosine sulfation is required for optimal function of mouse Psgl-1 in vivo and suggests that tyrosine sulfation of Psgl-1 contributes to the development of atherosclerosis.

## Introduction

Atherosclerosis is a chronic inflammatory disease of the arterial wall [1], [2]. It is initiated by vascular endothelial injury that leads to endothelial dysfunction and intramural accumulation of oxidized LDL. This causes the elaboration of signalling molecules and induction of adhesion receptors that promotes recruitment of monocytes into the vessel wall, a dominant factor in the initiation and progression of atherosclerosis [3].

We recently examined the importance of tyrosine sulfation in the development of atherosclerosis in a model in which lethally-irradiated *Ldlr*−/− mice were rescued with hematopoietic progenitors lacking tyrosylprotein sulfotransferase (TPST) activity [4]. We observed substantial reductions in aortic root lesion size and the number of macrophages in lesions in hyperlipidemic *Ldlr*−/− recipients transplanted with TPST deficient progenitors compared to controls. These data indicate that tyrosine sulfation of one or more proteins expressed in hematopoietic cells has a major impact on the development of atherosclerosis. The identities and the relative importance of the tyrosine-sulfated proteins involved are unknown. However, P-selectin glycoprotein ligand-1 (Psgl-1), along with the chemokine receptors Ccr2, Ccr5, and Cx3cr1 are likely candidates [4], [5].

Psgl-1 is a homodimeric mucin that is broadly expressed on hematopoietic cells [6]. In mice lacking P-selectin or Psgl-1, leukocyte rolling is virtually absent in a model of trauma induced P-selectin expression in post-capillary venules [7], [8]. Thus, Psgl-1 is the major physiologic ligand for P-selectin [9]. Psgl-1 is also a key player in the development of atherosclerosis. Psgl-1 expressed on Ly-6C^hi^ monocytes is a major mediator of monocyte recruitment into atherosclerotic lesions in mice, and aortic root lesions are ≈40% smaller in hyperlipidemic *ApoE*−/−; *Selplg*−/− mice compared to *ApoE*−/− mice [10]. In addition, transient P-selectin or Psgl-1 blockade using mAbs reduces macrophage influx and neointima formation in a model of arterial injury in *ApoE*−/− mice [11].

**Figure 1:**
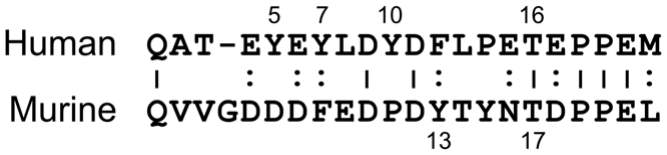
Sequence alignment of the N-terminal P-selectin binding sites of human and mouse P-selectin glycoprotein ligand-1.

Structure-function relationships for human PSGL-1 have been defined in great detail. The P-selectin binding site spans ≈15 residues near the N-terminus of the mature polypeptide, it contains sulfotyrosine residues at positions 5, 7, and 10 and a core 2 O-glycan terminating with sialyl-Le^x^ linked to Thr16 [12], [13], [14], [15]. Together these structural features are both necessary and sufficient for P-selectin binding. However, for mouse Psgl-1 the structure-function relationships are not as clearly defined. Like human PSGL-1, the P-selectin binding site of mouse Psgl-1 is near the N-terminus as defined by function blocking mAbs, but its amino acid sequence is considerably different than that of human PSGL-1 (Fig. 1) [16], [17].

Mouse Psgl-1 has two threonine residues (Thr14 and Thr17) near the N-terminus that are possible sites for O-glycan addition. P-selectin dependent rolling is severely impaired in fucosyltransferase VII (FucTVII) and core 2 β(1,6)-N-acetylglucosaminyltransferase-I (C2GlcNAcT-I) deficient mice indicating that mouse Psgl-1 requires α(1,3)fucosylated, core 2 O-glycan for optimal P-selectin binding in vivo [18], [19]. Furthermore, mouse Psgl-1 endogenously expressed in murine WEHI-3 cells carries core 2 O-glycan capped with sialyl-Le^x^ and site-directed mutagenesis studies implicate Thr17 as a site of O-glycan addition [20], [21].

In contrast to the substantial evidence for the importance of O-glycosylation, the importance of tyrosine sulfation of mouse Psgl-1 for P-selectin binding has not been definitely demonstrated. Indeed, it was only recently that mouse Psgl-1 was shown to be tyrosine-sulfated [4]. Tyr13 and Tyr15 are the only possible sulfation sites because they are the only tyrosine residues in the extracellular domain. The only study addressing the potential importance of tyrosine sulfation for mouse Psgl-1 is that of Xia et al who examined the function of recombinant mouse Psgl-1 expressed in Chinese hamster ovary (CHO) cells stably expressing human FucTVII and human C2GlcNAcT-I in vitro [21]. They observed that Tyr→Phe substitution at position 13, but not at position 15, impaired P-selectin binding and rolling of CHO cells on P-selectin in vitro, suggesting that Tyr13 was sulfated. However, the authors noted that their results should be interpreted cautiously because amino acid substitutions might impair function indirectly.

We therefore sought to directly examine the functional importance of tyrosine sulfation of native mouse Psgl-1 by testing its function in leukocytes lacking endogenous TPST activity in vivo. To accomplish this, mice were transplanted with hematopoietic progenitors from mice lacking both the *Tpst1* and *Tpst2* genes, the only TPSTs expressed in mice, and the rolling behaviour of TPST deficient leukocytes was examined in well-characterized, physiologically relevant assays. We found that TPST deficient leukocytes roll on P-selectin in vivo and in vitro. However, rolling of TPST deficient leukocytes was less efficient than wild type leukocytes despite equivalent surface expression of Psgl-1.

## Methods

### Ethics statement

All procedures involving vertebrate animals were reviewed and approved by the Institutional Animal Care and Use Committee at the Oklahoma Medical Research Foundation (Protocol #W0070).

### Antibodies

PE-conjugated anti-mouse Psgl-1 mAb 2PH1 (rat IgG_1_-κ), anti-mouse Psgl-1 mAb 4RA10 (rat IgG_1_-κ), FITC-CD45.1 mAb A20 (mouse IgG_2A_-κ), PE-CD45.2 mAb 104 (mouse IgG_2A_-κ), anti-mouse P-selectin mAb RB40.34 (rat IgG_1_-λ), and anti-mouse CD16/CD32 mAb 2.4G2 (rat IgG_2B_-κ, Mouse BD Fc Block™) were from BD Pharmingen. Anti-mouse Psgl-1 mAb 4RB12 (rat IgG_2A_) was provided by Dietmar Vestweber (Max Planck Institute for Molecular Biomedicine, Münster, Germany). Goat anti-mouse CD16/CD32 polyclonal antibody was from R&D Systems.

### Hematopoietic transplantation

*Tpst1*;*Tpst2* double knockout (*Tpst* DKO) mice were generated and characterized as previously described [22], [23]. These mice have severely impaired post-natal viability. Therefore, fetal livers were used as the source of hematopoietic progenitors. Lethally-irradiated B6.SJL-Ptprc^a^ Pep3^b^/BoyJ recipients (B6.SJL, The Jackson Laboratory, Stock #002014) were transplanted with E15.5 fetal liver cells from wild type 129S6 or *Tpst* DKO mice, which are in the 129S6 background as described previously [4]. These groups are abbreviated as WT→B6 and *Tpst* DKO→B6, respectively. All studies were conducted 16–24 weeks after transplantation. Complete blood counts were determined at the time of experimentation as previously described [4].

### Intravital microscopy

Mice were anesthetized, placed on a warmed microscope stage, and a catheter was placed in the left carotid artery for injections and blood sampling. Exteriorization of the cremaster muscle was used to induce P-selectin-dependent leukocyte rolling [24], [25]. The cremaster muscle was mounted on an observation portal and continuously bathed with Hank's balanced salt solution or 131.9 mM NaCl, 18 mM NaHCO_3_, 4.7 mM KCl, 2.0 mM CaCl_2_ and 2 mM MgSO_4_, pH 7.2 equilibrated with 79% N_2_ and 16% CO_2_ and 5% O_2_ at 36°C. All data collection was completed within 20 min of exteriorization of the cremaster muscle.

Observations of post-capillary venules were made using a Nikon Eclipse E600-FN microscope equipped with a water immersion objective (40x/0.80 W). Images were recorded using a CCD camera (DC-330E, Dage-MTI) and centerline velocities (v_CL_) were measured using an optical doppler velocimeter (Microvessel Velocity OD-RT, CircuSoft Instrumentation). Vessel diameter and the distance leukocytes rolled were determined from recorded images using a digital image processing system (SGI O2 workstation running Inovision ISEE® v5.24 software) and freeze-frame advancing.

Rolling flux fractions were calculated by dividing leukocyte rolling flux, defined as the number of rolling leukocytes passing a line perpendicular to the vessel axis over a period of 1 min, by total leukocyte flux estimated as WBC • v_b_ • π • (d/2)^2^, where WBC is total leukocyte count, v_b_ is mean blood flow velocity (v_CL_ • 0.625) and d is vessel diameter [26]. Rolling velocities for 10 leukocytes passing a line perpendicular to the vessel axis were measured in the same venules as rolling flux fractions. Leukocytes were analyzed for a period of 1 s (30 frames). Mean rolling velocity was calculated by dividing the distance travelled by the elapsed time.

Leukocyte interaction with the vessel wall was considered as rolling and not free flowing when velocities were below the critical velocity estimated as v_crit_  = v_b_ • ε • (2 - ε), where ε is the ratio of the leukocyte diameter to vessel diameter [27]. The leukocyte diameter is taken to be 7 µm [28]. Wall shear rates (γ_w_) were estimated as γ_w_  = 4.9 (8 v_b_ /d) where 4.9 is a correction factor obtained from velocity profiles determined using microparticle image velocimetry in microvessels [29], [30], [31].

### Parallel plate rolling assays

Polystyrene 35-mm dishes were coated with anti-human IgM Fc mAb (20 µg/ml, clone MH15-1, Accurate Chemical & Scientific) in HBSS overnight at 4°C. Dishes were washed with HBSS, 0.1% human serum albumin (HSA), blocked with HBSS, 1% HSA for 2 h and then incubated for 1 h at 37°C with media from COS-7 cells transfected with plasmids encoding mouse P-selectin/IgM or mouse CD45/IgM chimera. The plasmids were from by John B. Lowe (University of Michigan) and the conditioned media was kindly provided by Dr. Lijun Xia and John Michael McDaniel (Oklahoma Medical Research Foundation). P-selectin site densities were determined using ^125^I-labeled RB40.34 [32]. In some experiments dishes were pre-incubated with blocking P-selectin mAb RB40.34 (20 µg/ml, 1 h).

Bone marrow cells were flushed from femurs and passed through a 40 µm filter. Erythrocytes were lysed and cells were pelleted and resuspended in HBSS, 0.5% HSA at 0.5×10^6^ cells/ml. Cells were drawn through a parallel plate flow chamber (GlycoTech) using a PHD 200 syringe pump (Harvard Apparatus). Rolling leukocytes were observed using a Zeiss Axiovert 200 microscope equipped with a 20x/0.3 Ph1 objective. After 5 minutes, images were recorded using a CCD camera (XC-77, Hamamatsu Photonics) and analyzed using the image processing system described above. For each experiment, rolling leukocytes were analyzed in 4 fields in a vertical line perpendicular to the direction of flow. The number of rolling cells was converted to cells/mm^2^ and the mean rolling velocities of 10 cells in each field were calculated by dividing the distance travelled by the elapsed time.

### Flow cytometry

The degree of donor hematopoiesis in B6.SJL recipients (CD45.1^+^) transplanted with wild type or *Tpst* DKO (CD45.2^+^) hematopoietic cells was assessed by flow cytometry. Leukocytes were collected from bone marrow or peripheral blood into 2 mM EDTA. Erythrocytes were lysed and cells were pelleted and resuspended in HBSS, 1% FBS, 0.02% NaN_3_. Cells were incubated in mouse Fc block (10 µg/ml) followed by FITC-CD45.1 (10 µg/ml) and PE-CD45.2 (10 µg/ml). Following washing, cells were fixed with 1% paraformaldehyde in HBSS, 0.02% NaN_3_ and analyzed in a FACSCalibur™ flow cytometer (Becton Dickinson).

To quantitate Psgl-1 surface expression, cells were incubated with goat anti-mouse Fc block followed by PE-2PH1 (5 µg/ml) or 4RB12 (5 µg/ml) followed by FITC rabbit anti-rat IgG (10 µg/ml, Vector Labs). To assess P-selectin binding, cells were incubated with mouse P-selectin/IgM or control CD45/IgM and bound chimera was detected with FITC goat anti-human IgM (10 µg/ml, Chemicon). Analyses were gated on the neutrophil and monocyte population of donor (CD45.2^+^) origin. In some experiments, blocking mAbs against mouse P-selectin (RB40.34, 20 µg/ml) or mouse Psgl-1 (4RA10, 30 µg/ml) were included.

### Statistical Analysis

Differences in rolling velocity and rolling flux fraction were determined using independent samples *t*-tests using SPSS (SPSS for Mac, rel. 18.0). In addition to the *p*-values for the *t*-test, we present the effect size, Cohen's *d*, which measures the magnitude of the difference between the two group means expressed in terms of standard deviation. A Cohen's *d* value ≥0.8 represents a large effect size [33]. All tests were two-tailed and an α ≤0.05 was set for statistical significance. All results are represented as the mean ± S.E.M.

## Results

### Hematopoietic reconstitution

To assess the efficiency of reconstitution of *Tpst* DKO hematopoiesis in B6.SJL recipients, complete blood counts and the percentage of donor (CD45.2^+^) cells were determined at the time of experimentation at 16–24 weeks after transplantation. We observed that the total leukocyte, neutrophil, lymphocyte, monocyte, erythrocyte and platelet counts were normal and that there were no significant differences between the two transplant groups (Table 1).

**Table 1:** Complete blood counts.

Experimental Group	Leukocytes (×10^−3^/µl)	Neutrophils (×10^−3^/µl)	Lymphocytes (×10^−3^/µl)	Monocytes (×10^−3^/µl)	Erythrocytes (×10^−6^/µl)	Platelets (×10^−3^/µl)
WT→B6	4.87±0.76	1.28±0.17	3.21±0.57	0.30±0.08	8.38±0.25	645±58
*Tpst* DKO→B6	5.64±0.70	1.25±0.19	3.98±0.47	0.30±0.08	7.82±0.28	632±56
*p*-value	0.461	0.896	0.304	0.973	0.152	0.872

Complete blood counts 16–24 weeks post-transplant are expressed as the mean ± S.E.M. (n = 13). Statistical differences between the groups were assessed using a Student's two-tailed *t*-test with unequal sample variance.

For in vivo rolling and P-selectin binding studies using peripheral blood, 96.2±0.7% (n = 10) of circulating neutrophils and monocytes in WT→B6 mice and 94.7±1.2% (n = 17) in *Tpst* DKO→B6 mice were CD45.2^+^ at the time of experimentation. For in vitro rolling and P-selectin binding studies using bone marrow leukocytes, 97.4±0.5% (n = 4) of bone marrow leukocytes in WT→B6 mice and 96.5±1.2% (n = 6) in *Tpst* DKO→B6 mice were CD45.2^+^ at the time of experimentation.

Mice appeared normal with no clinical signs of graft vs. host disease (i.e. diarrhea). Furthermore, body weights of the WT→B6 group (22.0±0.4 g, n = 13) were similar to the *Tpst* DKO→B6 group (23.1±0.5 g, n = 13). Taken together, these data demonstrate efficient reconstitution of donor hematopoiesis in the B6.SJL recipients, confirming the histocompatibility of the donor-recipient pair.

### P-selectin-dependent leukocyte rolling in vivo

**Figure 2:**
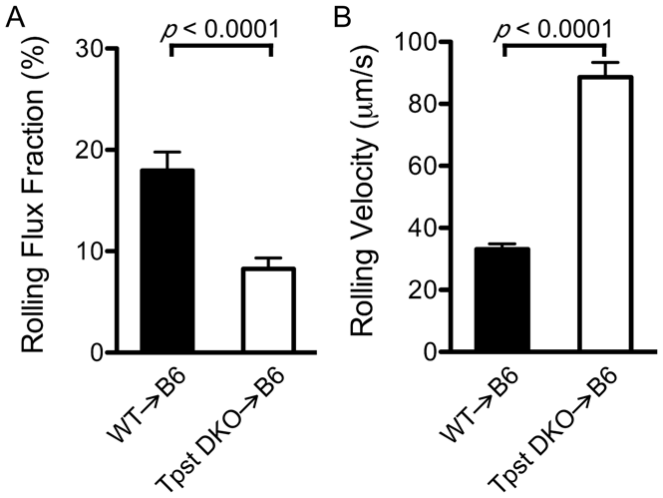
P-selectin dependent rolling in vivo. Surgical exteriorization of the cremaster muscle was used to induce P-selectin-dependent leukocyte rolling in post-capillary venules. (A) The rolling flux fraction in 49 venules from 7 WT→B6 mice and 39 venules from 7 *Tpst* DKO→B6 mice was determined. (B) Mean rolling velocities were determined from an average of 8.5 leukocytes/venule in the same venules as the rolling flux fractions. All values are reported as mean ± S.E.M.

P-selectin-dependent leukocyte rolling in post-capillary venules was induced by exteriorization of the cremaster muscle. Rolling flux fractions were quantitated in 49 venules in 7 WT→B6 mice and 39 venules in 7 *Tpst* DKO→B6 mice and leukocyte rolling velocities were determined for an average of 8.5 rolling cells in each venule (Fig. 2A and B). Hemodynamic and microvascular parameters including venule diameter, centerline velocity and wall shear rates were comparable in WT→B6 and *Tpst* DKO→B6 mice (Table 2).

**Table 2:** Hemodynamic and microvascular parameters.

Experimental Group	Mice(n)	Venules(n)	Diameter(µm)	Centerline velocity(µm/s)	Wall shear rate(s^−1^)
WT→B6	7	49	30.0±1.0	1369±86	949±66
*Tpst* DKO→B6	7	39	29.9±1.1	1123±85	1229±104

Values are expressed as the mean ± S.E.M.

We observed that rolling flux fractions were lower in *Tpst* DKO→B6 venules (8.3±1.1%, n = 39 venules) compared to WT→B6 venules (18.0±1.8%, n = 49 venules). We also observed that leukocyte rolling velocities were higher in *Tpst* DKO→B6 venules (88.6±4.8 µm/s) compared to WT→B6 venules (33.1±1.8 µm/s). Statistical analysis showed that rolling flux fraction in *Tpst* DKO→B6 venules were significantly lower (*p*<0.0001, *d* = 0.96) and rolling velocities were significantly higher in the *Tpst* DKO→B6 group compared to the WT→B6 group (*p*<0.0001, *d* = 2.4).

In some experiments, blocking mAbs to P-selectin or Psgl-1 were administered after initial data collection and venules were re-examined for rolling leukocytes. In each of these experiments observations were made before and immediately after mAb administration in a single venule and then in an additional 2–7 venules in each animal. We observed that rolling leukocytes, defined as those with velocities less than v_crit_, were undetectable after administration of 10 µg of P-selectin mAb RB40.34 in both WT→B6 and *Tpst* DKO→B6 mice (n = 3). In a separate series of experiments, rolling leukocytes were also undetectable in WT→B6 and *Tpst* DKO→B6 mice (n = 3) after administration of 10 µg of Psgl-1 mAb 4RA10.

### P-selectin-dependent leukocyte rolling in vitro

To study P-selectin-dependent rolling in a more defined system, leukocyte rolling was observed on P-selectin coated dishes in a parallel plate flow chamber. The number of rolling leukocytes and leukocyte rolling velocities were quantitated in three independent experiments comparing leukocytes harvested from wild type and *Tpst* DKO→B6 mice. Bone marrow leukocytes were harvested and drawn over dishes coated with mouse P-selectin/IgM at a shear stress of 1 dyn/cm^2^ as described in Methods.

**Figure 3:**
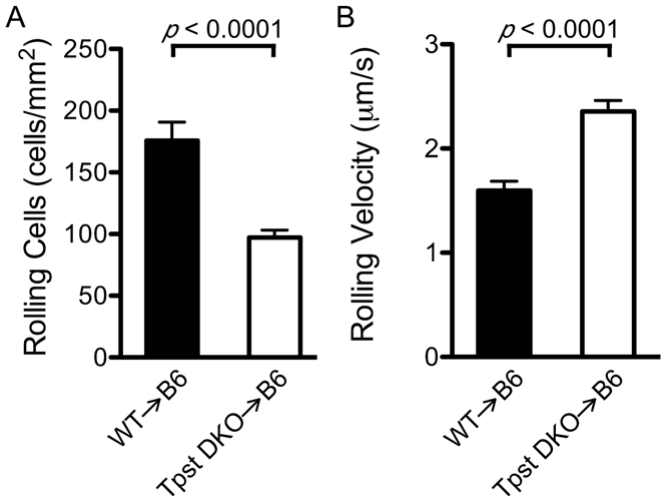
P-selectin dependent leukocyte rolling in vitro. Bone marrow leukocytes from wild type (n = 3) and *Tpst* DKO→B6 (n = 3) mice were isolated and their rolling on immobilized mouse P-selectin/IgM (site density  = 100 sites/µm^2^) was observed at 1 dyn/cm^2^. (A) For each animal, the number of rolling cells in 4 fields of view were averaged. (B) Rolling velocities of 10 leukocytes were determined in the same 4 fields of view as the number of rolling cells. Values are reported as mean ± S.E.M.

For wild type leukocytes, we observed 176±15 rolling cells/mm^2^, whereas for *Tpst* DKO→B6 leukocytes we observed only 97±6 rolling cells/mm^2^ (n = 12 fields from 3 independent paired experiments) (Fig. 3A). Statistical analysis showed that the number of rolling cells in the *Tpst* DKO→B6 group was significantly lower than the WT group (*p*<0.0001, *d* = 2.1).

In the same experiments, the velocities of 10 individual leukocytes in each of the 4 fields observed were measured. We found that wild type leukocytes rolled with a mean velocity of 1.6±0.1 µm/s. In contrast, *Tpst* DKO→B6 leukocytes had mean rolling velocities of 2.4±0.1 µm/s (Fig. 3B). Statistical analysis showed that rolling velocities were significantly higher in the *Tpst* DKO→B6 group compared to the WT group (*p*<0.0001, *d* = 2.3). No detectable leukocyte rolling was observed on dishes coated with mouse CD45/IgM or when dishes coated with P-selectin/IgM were pre-incubated with the P-selectin blocking mAb RB40.34 (data not shown).

### P-selectin binding and Psgl-1 expression

**Figure 4:**
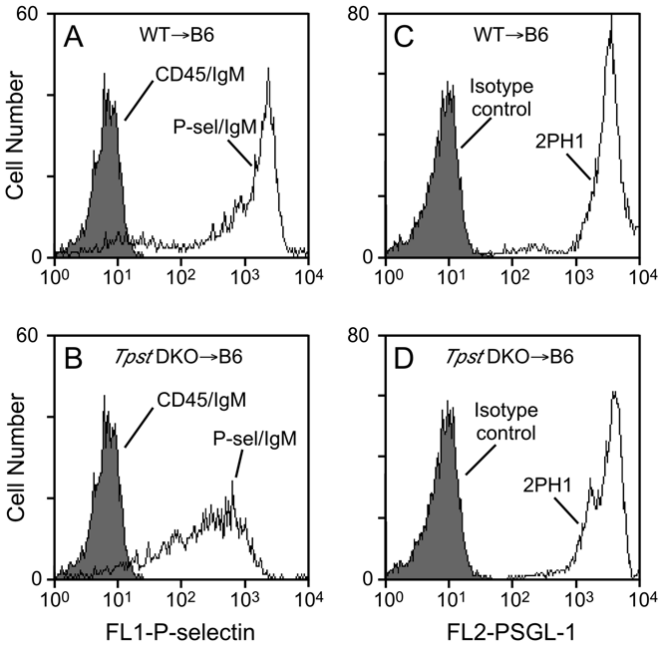
Binding of fluid-phase P-selectin and Psgl-1 expression. Binding of P-selectin/IgM to peripheral blood leukocytes from (A) WT→B6 mice or (B) *Tpst* DKO→B6 mice. Shaded histograms represent binding of CD45/IgM. Binding of the anti-Psgl-1 mAb 2PH1 to peripheral blood leukocytes from (C) WT→B6 mice or (D) *Tpst* DKO→B6 mice. Shaded histograms represent binding of isotype control mAb. Panels A & C are same samples analyzed on the same day and are representative of 3 WT→B6 mice. Panels B & D are also same samples analyzed on the same day and are representative of 7 *Tpst* DKO→B6 mice. All analyses were gated on the neutrophil and monocyte population based on forward and orthogonal light scattering properties and on donor origin (CD45.2^+^).

P-selectin binding to Psgl-1 on neutrophils in peripheral blood was determined using flow cytometry. Neutrophils were gated based on their forward and orthogonal light scattering properties and on donor origin (CD45.2^+^). We observed that the mean fluorescence intensity (MFI) of P-selectin binding to WT→B6 cells was 1,322±138 (n = 3) and 557±64 for *Tpst* DKO→B6 cells (n = 7) (Fig. 4A & B). This difference is highly significant (*p* = 0.016). Pre-incubation of cells with P-selectin blocking mAb RB40.34 or Psgl-1 blocking mAb 4RA10 completely blocked binding of the P-selectin/IgM to levels equivalent to that for CD45/IgM (data not shown).

These samples were also analyzed by flow cytometry using the Psgl-1 blocking mAb 2PH1, to investigate whether the reduced rolling of *Tpst* DKO cells and reduced binding of P-selectin to *Tpst* DKO neutrophils was due to altered surface expression of Psgl-1. We observed that MFI of 2PH1 binding to neutrophils from WT→B6 mice (3,488±169, n = 3) and *Tpst* DKO→B6 mice were indistinguishable (3,322±123, n = 7, *p* = 0.47) (Fig. 4C & D). We also examined Psgl-1 expression on leukocytes from a rare *Tpst* DKO mouse at post-natal day 14 using 4RB12, a non-blocking antibody to Psgl-1. 4RB12 binding to peripheral blood neutrophils and bone marrow leukocytes from the *Tpst* DKO mouse was indistinguishable from an age-matched wild type mouse examined in parallel (data not shown).

## Discussion

We recently reported that transplantation of *Ldlr*−/− mice with *Tpst* DKO hematopoietic progenitors drastically attenuated development of atherosclerosis [4]. This result indicated that tyrosine sulfation of one or more proteins expressed in hematopoietic cells has a major impact on the development of atherosclerosis. Psgl-1 is one likely candidate because it is known to be tyrosine-sulfated in the mouse and its role in monocyte recruitment in atherosclerosis is well established [4], [10], [11]. We therefore sought to directly examine the functional importance of tyrosine sulfation for Psgl-1 in vivo.

To address this question, mice were transplanted with hematopoietic progenitors from mice lacking endogenous TPST activity and the rolling behaviour of TPST deficient leukocytes was examined in a well-characterized model of trauma induced P-selectin expression in post-capillary venules in the cremaster muscle. We observed that significantly fewer TPST deficient leukocytes rolled in post-capillary venules in the *Tpst* DKO→B6 group compared to wild type leukocytes in the WT→B6 group and TPST deficient leukocytes rolled at significantly higher velocities than wild type leukocytes. These observations were confirmed in two well-defined in vitro assay systems. First, in a parallel plate adhesion assay, fewer TPST deficient leukocytes rolled on P-selectin and they rolled at higher velocities compared to wild type leukocytes under physiologically relevant shear stress. In addition, in a flow cytometry assay, binding of fluid-phase P-selectin to TPST deficient peripheral blood and bone marrow leukocytes was significantly lower than binding to wild type leukocytes. Importantly, we showed that impaired rolling in vivo and in vitro and impaired binding of fluid-phase P-selectin to TPST deficient leukocytes was not due to differences in surface expression of Psgl-1. Finally, antibody blocking experiments in *Tpst* DKO→B6 mice showed that anti-Psgl-1 mAb abolished P-selectin dependent rolling in vivo. Taken together these observations demonstrate that tyrosine sulfation enhances the binding capacity of mouse Psgl-1, but is not an absolute requirement for Psgl-1 function in vivo. These findings, in conjunction with our previous report that atherosclerosis is attenuated in hyperlipidemic *Ldlr*−/− mice with *Tpst* DKO hematopoiesis, suggest that tyrosine sulfation of Psgl-1 may contribute to lesion development.

It is formally possible that impaired rolling of TPST deficient leukocytes is due to differences in O-glycosylation of Psgl-1 compared to wild type leukocytes. However, it is difficult to envision how O-glycosylation, that occurs in an earlier Golgi compartment, could be impacted by the presence or absence of tyrosine sulfation that occurs in the trans-Golgi network [34], [35].

In our studies, leukocyte rolling was completely abrogated by injection of a blocking P-selectin antibody. This is consistent with published data that leukocyte rolling in this model is entirely dependent on P-selectin expression on the post-capillary venules [25]. Psgl-1 is the predominant ligand for P-selectin is the early phases (<30 min) after trauma-induced inflammation in the mouse cremaster. However, previous studies indicate that a minor component of P-selectin-dependent rolling in this model is Psgl-1-independent [16], [19], [36], [37]. For example, Yang et al reported that rolling flux fraction was severely reduced but detectable in Psgl-1 deficient animals (1.2%) compared wild type controls (20.9%) [8]. In addition, Sperandio et al reported that administration of the anti-Psgl-1 mAb 4RA10 to wild type mice reduced rolling flux fraction from 27% to 8% and increased rolling velocities from 44 to 110 µm/sec. In our study, rolling was abolished by 4RA10 in both WT→B6 and *Tpst* DKO→B6 mice. Thus, we do not detect a Psgl-1-independent component of P-selectin-dependent rolling that has been reported by others.

Our findings provide strong support for previous in vitro observations by Xia et al, who examined the effects of site-directed mutagenesis and sodium chlorate on mouse Psgl-1 function in CHO cells stably expressing human FucT-VII and C2GlcNAcT-I [21]. Sodium chlorate inhibits synthesis of the sulfate donor PAPS and therefore blocks the action of all sulfotransferases [38]. They reported that mutagenesis of Tyr13, but not Tyr15, to Phe or incubation of cells with sodium chlorate impaired, but did not abolish P-selectin binding and rolling of the transfected CHO cells. Although this implicates Tyr13 as a potential site for sulfation, these observations do not prove that Tyr13 is sulfated and that Tyr15 is not, because Tyr to Phe substitution(s) might impair function indirectly by altering the protein conformation or may affect sulfate addition at the nearby non-mutated tyrosine. Thus, further studies are necessary to directly determine the precise location and stoichiometry of sulfation.

In summary, we examined the functional role for tyrosine sulfation of mouse Psgl-1 using physiologically relevant assay systems in a unique model in which mice were transplanted with hematopoietic progenitors from mice lacking TPST activity. This model enabled examination of mouse Psgl-1 function in a native mouse leukocyte modified by endogenous mouse glycosyltransferases without altering the amino acid sequence of the protein. Our studies provide direct and convincing evidence that tyrosine sulfation is required for optimal function of mouse Psgl-1 in vivo.
